# Effective Gibbs State for Averaged Observables

**DOI:** 10.3390/e24081144

**Published:** 2022-08-18

**Authors:** Alexander Evgen’evich Teretenkov

**Affiliations:** Department of Mathematical Methods for Quantum Technologies, Steklov Mathematical Institute of Russian Academy of Sciences, ul. Gubkina 8, Moscow 119991, Russia; taemsu@mail.ru

**Keywords:** effective Hamiltonian, Gibbs state, quantum thermodynamics

## Abstract

We introduce the effective Gibbs state for the observables averaged with respect to fast free dynamics. We prove that the information loss due to the restriction of our measurement capabilities to such averaged observables is non-negative and discuss a thermodynamic role of it. We show that there are a lot of similarities between this effective Hamiltonian and the mean force Hamiltonian, which suggests a generalization of quantum thermodynamics including both cases. We also perturbatively calculate the effective Hamiltonian and correspondent corrections to the thermodynamic quantities and illustrate it with several examples.

## 1. Introduction

There are a lot of physical models which use averaging with respect to fast oscillations one way or another. For example, many derivations of master equations use secular approximation directly ([1] Subsection 3.3.1), ([2] Section 5.2) or as result [3,4] of perturbation theory with Bogolubov–van Hove scaling [5,6] (see also corrections beyond the zeroth order in [7]). Moreover, there is a wide discussion of the applicability of the rotating wave approximation (RWA) and the systematic perturbative corrections to it in the literature [8,9,10,11,12,13,14,15,16,17]. However, in this work, we consider such averaging not as an approximation but as a restriction of our observation capabilities. In addition, we analyze the thermodynamic equilibrium properties of a quantum system, assuming such restrictions. Due to this averaging, the thermodynamic equilibrium properties can be defined by some effective Gibbs state, which is averaged with respect to these fast oscillations, instead of the exact Gibbs state. Similarly to strong coupling thermodynamics, this effective Gibbs state can be defined by some effective temperature-dependent Hamiltonian, which is an analog of the mean force Hamiltonian (see, e.g., ([18] Chapter 22), [19,20] for recent reviews).

In Section 2, we describe the setup of our problem and develop a systematic perturbative calculation for the effective Hamiltonian. We show that the zeroth and the first term of the expansion coincide with the RWA Hamiltonian and, in particular, are temperature independent. In this point, it is similar to effective Hamiltonians also arising as corrections to the RWA but in dynamical and non-equilibrium problems. The second-order term is temperature-dependent. We show that both this term and its derivative with respect to the inverse temperature are non-positive definite.

In Section 3, we show that this definiteness is closely related to the positivity of the information loss due to the fact that we have access only to the averaged observables discussed above rather than all possible observables. We show that information loss leads to energy loss, which is hidden from our observation. We prove (without perturbation theory) that these losses are always non-negative, but in the leading order, they are defined by the second-order temperature-dependent term in the effective Hamiltonian expansion. Additionally, we prove that exact non-equilibrium free energy is always larger than the free energy observable in our setup. If one assumes that the effective Gibbs state is an exact state, then this difference is also defined by the second-order term of the effective Hamiltonian expansion. At the end of Section 3, we argue that the analogy between our effective Hamiltonian and the mean force Hamiltonian is because they are special cases of the general setup, based on so-called conditional expectations.

To dwell on this analogy, in Section 4, we consider a compound system and the mean force Hamiltonian of one of the subsystems for the effective Gibbs state discussed above. We also give the systematic perturbative expansion for it.

In Section 5, we consider several simple examples to illustrate the results of the previous sections. Namely, we consider two interacting two-level systems, two interacting oscillators and a two-level system interacting with the oscillator. We calculate the effective Hamiltonians for such systems and the information losses due to the restriction to the averaged observables.

Both the effective Hamiltonian we define in this work and the explicit perturbative expansion for it are novel, but such a Hamiltonian has much in common with the mean force Hamiltonian (see the end of Section 3 for a more precise discussion). The main difference consists of the choice of a projector. Thus, our results suggest the possibility to generalize equilibrium quantum thermodynamics to effective equilibrium quantum thermodynamics by different choices of the projector.

## 2. Effective Hamiltonian

We are interested in equilibrium properties of fast oscillating observables which are in resonance with the free Hamiltonian. We assume that the equilibrium state has the Gibbs form

(1) $$\rho_{\beta }=\frac{e^{-\beta H}}{Z}$$

with inverse temperature β>0 and the Hamiltonian of the form

(2) $$H=H_{0}+\lambda H_{I},$$

where $H_{0}$ is a free Hamiltonian and $H_{I}$ is an interaction Hamiltonian, λ is a small parameter.

In addition, we consider the observables which are explicitly time-dependent with very specific time dependence. Namely, they depend on time in the Schrödinger picture as follows

(3) $$X\left(t\right)=e^{-iH_{0}t}Xe^{iH_{0}t}$$

i.e., they depend on time in such a way that they become constant in the interaction picture for the “free” Hamiltonian $H_{0}$. A widely used example of such an observable is a dipole operator interacting with the classical electromagnetic field in resonance with a free Hamiltonian (see, e.g., [21] Section 15.3.1). In addition, we assume that one could actually observe the long-time averages

(4) $${\langle X\left(t\right)\rangle }_{\mathrm{av}}=\lim_{T\to +\infty }\frac{1}{T}\int_{0}^{T}\langle X\left(t\right)\rangle dt,$$

where $\langle X\left(t\right)\rangle \equiv \mathrm{Tr}\rho_{\beta }X\left(t\right)$. By “long”, we mean long with respect to inverses of non-zero Bohr frequencies, where Bohr frequencies are the eigenvalues of the superoperator $\left[H_{0},\;\cdot \;\right]$ (see, e.g., [4] p. 122). The observation of such long-time averages is usual for spectroscopy setups ([22] Section 4). Moreover, we will further discuss the perturbation theory in λ, assuming that this averaging is already performed, so this long timescale remains “long” even being multiplied by any power of λ. Otherwise, one should introduce the small parameter in the averaging procedure as well, which leads to more complicated perturbation theory depending on how the small parameter in the averaging and in the Hamiltonian are related to each other.

Average (4) can be represented as

(5) $${\langle X\left(t\right)\rangle }_{\mathrm{av}}=\mathrm{Tr}X{\tilde{\rho }}_{\beta },$$

where ${\tilde{\rho }}_{\beta }$ is some effective Gibbs state, which could be calculated as

(6) $${\tilde{\rho }}_{\beta }=P\rho_{\beta },$$

where

(7) $$PX=\lim_{T\to +\infty }\frac{1}{T}\int_{0}^{T}e^{iH_{0}t}Xe^{-iH_{0}t}dt,$$

because

(8) $$\begin{array}{cc} {\langle X\left(t\right)\rangle }_{\mathrm{av}} & =\lim_{T\to +\infty }\frac{1}{T}\int_{0}^{T}\mathrm{Tr}e^{-iH_{0}t}Xe^{iH_{0}t}\rho_{\beta }dt\\ \; & =\lim_{T\to +\infty }\frac{1}{T}\int_{0}^{T}\mathrm{Tr}Xe^{iH_{0}t}\rho_{\beta }e^{-iH_{0}t}dt=\mathrm{Tr}XP\rho_{\beta }. \end{array}$$

From the thermodynamical point of view, it is natural to represent this effective Gibbs state in the Gibbs-like form

(9) $${\tilde{\rho }}_{\beta }=\frac{1}{Z}e^{-\beta \tilde{H}}$$

with some effective Hamiltonian $\tilde{H}$ similarly to the mean force Hamiltonian ([18], Chapter 22). Let us remark that we have the same partition function for both exact and effective Hamiltonians due to the fact that P is a trace-preserving map (see Appendix A) $\mathrm{Tr}e^{-\beta \tilde{H}}=\mathrm{Tr}Pe^{-\beta H}=\mathrm{Tr}e^{-\beta H}$. Let us summarize several properties of the superoperator P which will be used further (see Appendix A for the proof).

1. P is completely positive.
2. P is a self-adjoint (with respect to trace scalar product $\mathrm{Tr}X^{†}Y$) projector
  (10) $$P^{2}=P=P^{*}.$$
3. Let the spectral decomposition of $H_{0}$ have the form $H_{0}=\sum_{\varepsilon }\varepsilon \Pi_{\varepsilon }$, where ε are (distinct) eigenvalues of $H_{0}$ and $\Pi_{\varepsilon }$ are orthogonal projectors $\Pi_{\varepsilon }\Pi_{\varepsilon^{\prime }}=\delta_{\varepsilon \varepsilon^{\prime }}\Pi_{\varepsilon }$, $\Pi_{\varepsilon }=\Pi_{\varepsilon }^{†}$. Then,
  (11) $$PX=\sum_{\varepsilon }\Pi_{\varepsilon }X\Pi_{\varepsilon }$$
  for any matrix *X*.

For the case of one-dimensional projectors $\Pi_{\varepsilon }$, superoperator (11) is sometimes called the dephasing operation [23]. In the general case, it is usually called pinching [24], p. 16. It can also be understood as a special case of twirling [25] (with one-parameter group).

Effective Hamiltonian $\tilde{H}$ can be calculated by cumulant-type expansion. Namely, we have the following proposition (see Appendix B for the proof).

**Proposition** **1.** *The perturbative expansion of $\tilde{H}$ has the form*

(12) $$\tilde{H}=H_{0}-\beta^{-1}\sum_{n=1}\lambda^{n}\sum_{k_{0}+\cdots +k_{m}=n}\frac{{\left(-1\right)}^{m}}{m+1}M_{k_{0}}\left(\beta \right)M_{k_{1}}\left(\beta \right)\cdots M_{k_{m}}\left(\beta \right),$$

*where*

(13) $$M_{k}\left(\beta \right)={\left(-1\right)}^{k}\int_{0}^{\beta }d\beta_{1}\ldots \int_{0}^{\beta_{k-1}}d\beta_{k}PH_{I}\left(\beta_{1}\right)\ldots H_{I}\left(\beta_{k}\right)$$

*and*

(14) $$H_{I}\left(\beta \right)\equiv e^{\beta H_{0}}H_{I}e^{-\beta H_{0}}.$$

In particular, the first terms of the expansion have the form

(15) $$\tilde{H}=H_{0}-\beta^{-1}\lambda M_{1}\left(\beta \right)-\beta^{-1}\lambda^{2}\left(M_{2}\left(\beta \right)-\frac{1}{2}{\left(M_{1}\left(\beta \right)\right)}^{2}\right)+O\left(\lambda^{3}\right).$$

To make this expansion more explicit, let us represent the interaction Hamiltonian in the eigenbasis of the superoperator $\left[H_{0},\;\cdot \;\right]$ in the same way as it is usually performed for Markov master equation derivation ([1] Subsection 3.3.1)

(16) $$H_{I}=\sum_{\omega }D_{\omega },$$

where sum is taken over the Bohr frequencies and

(17) $$\left[H_{0},D_{\omega }\right]=-\omega D_{\omega }.$$

Moreover, as $H_{I}$ is Hermitian, then $D_{-\omega }=D_{\omega }^{†}$. Hence, we have the following explicit expressions for $M_{k}\left(\beta \right)$.

**Proposition** **2.** *If Equations (16) and (17) are held, then*

(18) $$M_{k}\left(\beta \right)={\left(-1\right)}^{k}\sum_{\omega_{1},\ldots ,\omega_{k-1}}g_{k}\left(\beta ;\omega_{1},\cdots ,\omega_{k-1}\right)D_{\omega_{1}}\cdots D_{\omega_{k-1}}D_{-\omega_{1}-\ldots -\omega_{k-1}},$$

*where*

(19) $$\begin{array}{cc} g_{k}(\beta ;\omega_{1}, & \ldots ,\omega_{k-1})=\frac{1}{\prod_{k=1}^{n-1}\sum_{j=1}^{k}\omega_{j}}\left(\beta -\sum_{k=1}^{n-1}\frac{1}{\sum_{j=1}^{k}\omega_{j}}\right)\\ \; & -\sum_{p=1}^{n-1}\frac{{\left(-1\right)}^{p}}{\left(\prod_{m=2}^{p}\sum_{i=m}^{p}\omega_{i}\right){\left(\sum_{r=1}^{p}\omega_{r}\right)}^{2}\left(\prod_{k=p+1}^{n-1}\sum_{j=p+1}^{k}\omega_{j}\right)}e^{-\beta \sum_{i=1}^{p}\omega_{i}}. \end{array}$$

*For zero denominators, it should be understood as a limit.*

The proof can be found in Appendix C. The first terms of expansion (15) take the form (see Appendix C)

(20) $$\tilde{H}=H_{0}+\lambda D_{0}-\lambda^{2}\sum_{\omega \neq 0}\frac{\beta \omega +e^{-\beta \omega }-1}{\beta \omega^{2}}D_{\omega }D_{\omega }^{†}+O\left(\lambda^{3}\right).$$

Thus, the first two terms are temperature-independent and recover the Hamiltonian in the rotating wave approximation (similarly to effective Hamiltonians for dynamical evolution [26,27])

(21) $$H_{\mathrm{RWA}}=H_{0}+\lambda D_{0}.$$

On the other hand, the next term of expansion (20) is the first temperature-dependent correction to the RWA Hamiltonian. This term is always non-positive definite

(22) $${\tilde{H}}^{\left(2\right)}\equiv -\sum_{\omega \neq 0}\frac{\beta \omega +e^{-\beta \omega }-1}{\beta \omega^{2}}D_{\omega }D_{\omega }^{†}\leq 0$$

due to the fact that it has the form

(23) $${\tilde{H}}^{\left(2\right)}=-\frac{\beta }{2}\sum_{\omega \neq 0}f\left(\beta \omega \right)D_{\omega }D_{\omega }^{†},$$

where $\langle \psi |D_{\omega }D_{\omega }^{†}|\psi \rangle =\left|\right|D_{\omega }^{†}{\left|\psi \rangle |\right|}^{2}\geq 0$ for arbitrary |ψ〉,

(24) $$f\left(x\right)\equiv 2\frac{x+e^{-x}-1}{x^{2}}$$

is a positive function f(x)>0 for all real *x* and β is assumed to be positive as we consider the positive temperature (but if one considers a negative temperature, which is possible for finite-dimensional systems, then ${\tilde{H}}^{\left(2\right)}$ becomes non-negative). Moreover, ${\tilde{H}}^{\left(2\right)}$ is a monotone function of temperature, because

(25) $$\frac{\partial }{\partial \beta }{\tilde{H}}^{\left(2\right)}=-\frac{1}{2}\sum_{\omega \neq 0}f_{1}\left(\beta \omega \right)D_{\omega }D_{\omega }^{†}\leq 0,$$

where

(26) $$f_{1}\left(x\right)\equiv 2\frac{1-e^{-x}\left(1+x\right)}{x^{2}}$$

is also a positive function for all real *x*. In the next section, we will see that if one averages this result with respect to the effective Gibbs state, then this result becomes closely related to general thermodynamic properties which are valid in all the orders of perturbation theory.

Let us also remark that $\lim_{x\to +0}f\left(x\right)=1$, so for the low temperature limit, i.e., when βω≫1 for all non-zero Bohr frequencies, Equation (23) takes the form

(27) $${\tilde{H}}^{\left(2\right)}\approx -\frac{\beta }{2}\sum_{\omega \neq 0}D_{\omega }D_{\omega }^{†},$$

i.e., the second-order correction in λ is linear in β.

In the recent literature, there is also rising interest in the ultrastrong coupling limit. Let us remark that ${\tilde{H}}^{\left(2\right)}$ is also the leading order difference between effective Hamiltonians for steady states for the ultrastrong coupling limit conjectured in [28] and the one obtained in [29], if one takes the interaction Hamiltonian as a free Hamiltonian in our notation and vice versa. The perturbative corrections for such steady states are discussed in [30].

## 3. Effective Hamiltonian as Analog of Mean Force Hamiltonian

The free energy *F* can be defined by the partition function *Z* as

(28) $$F=-\beta^{-1}\ln Z,$$

where, as it was mentioned before, *Z* could be defined by the same formula $Z=\mathrm{Tr}e^{-\beta H}=\mathrm{Tr}e^{-\beta \tilde{H}}$ both by exact Hamiltonian *H* and by effective Hamiltonian $\tilde{H}$. If one calculates the entropy and the internal energy by equilibrium thermodynamics formulae

(29) $$S=\beta^{2}\frac{\partial F}{\partial \beta },\;U=\frac{\partial (\beta F)}{\partial \beta },$$

then it also obviously does not matter if we use the exact or effective Hamiltonian. For initial temperature-independent Hamiltonian, they also could be calculated as:

(30) $$S=-\mathrm{Tr}\rho_{\beta }\ln\rho_{\beta },\;U=\mathrm{Tr}H\rho_{\beta }.$$

However, for the effective Hamiltonian, the similar formulae need additional corrections due to its dependence on temperature. Namely,

(31) $$S=\tilde{S}-\Delta S,\;U=\tilde{U}-\Delta U,$$

where $\tilde{S}$ and $\tilde{U}$ are defined by the formulae similar to Equation (30)

(32) $$\tilde{S}=-\mathrm{Tr}{\tilde{\rho }}_{\beta }\ln{\tilde{\rho }}_{\beta },\;\tilde{U}=\mathrm{Tr}\tilde{H}{\tilde{\rho }}_{\beta }$$

In addition, the corrections have exactly the same form as for the mean force Hamiltonian (see, e.g., [31], Equations (11) and (12))

(33) $$\Delta S=-\beta^{2}{\langle \partial_{\beta }\tilde{H}\rangle }_{∼},\;\Delta U=-\beta {\langle \partial_{\beta }\tilde{H}\rangle }_{∼}=\beta^{-1}\Delta S.$$

Here, ${\langle \;\cdot \;\rangle }_{∼}$ denotes the average with respect to the effective Gibbs state, i.e., ${\langle \;\cdot \;\rangle }_{∼}\equiv \mathrm{Tr}\left(\;\cdot \;{\tilde{\rho }}_{\beta }\right)$. The derivation of these formulae is exactly the same as for analogous formulae for the mean force Hamiltonian (see ([18], Chapter 22), [32]), because it is valid for an arbitrary temperature-dependent Hamiltonian and is based only on the Feynman–Wilcox formula [33,34,35]

(34) $$\frac{d}{d\beta }e^{-\beta \tilde{H}}=-\int_{0}^{t}dse^{-\left(1-s\right)\beta \tilde{H}}\left(\frac{d}{d\beta }\left(\beta \tilde{H}\right)\right)e^{-s\beta \tilde{H}}.$$

Due to the fact that P is a completely positive trace preserving and unital map (PI=I), the entropy is monotone [36], p. 136 under its action, i.e., $\tilde{S}\geq S$. Thus, ΔS≥0 and $\Delta U=\beta^{-1}\Delta S\geq 0$. $\tilde{S}$ and $\tilde{U}$ could be interpreted as entropy and as energy which are accessible to our observations. Our observable entropy is $\tilde{S}$, but due to our restricted observational capabilities, we have the information loss quantified by ΔS. This information loss comes with energy loss quantified by ΔU and is hidden from our observations.

For second-order expansion in λ, we have

(35) $$\Delta S=-\lambda^{2}\beta^{2}{\langle \partial_{\beta }{\tilde{H}}^{\left(2\right)}\rangle }_{∼}+O\left(\lambda^{3}\right)=-\lambda^{2}\beta^{2}{\langle \partial_{\beta }{\tilde{H}}^{\left(2\right)}\rangle }_{0}+O\left(\lambda^{3}\right),$$

where ${\langle \;\cdot \;\rangle }_{0}$ is the average with respect to the Gibbs state for the free Hamiltonian. Thus, the non-negativity of ΔS in the second order of perturbation theory agrees with Equation (25). Moreover, it could be calculated (see Appendix D) by the following formula

(36) $$\Delta S=-\lambda^{2}\beta {\langle {\tilde{H}}^{\left(2\right)}\rangle }_{0}+O\left(\lambda^{3}\right)=\sum_{\omega >0}\frac{1-e^{-\beta \omega }}{\beta \omega }{\langle D_{\omega }D_{\omega }^{†}\rangle }_{0}+O\left(\lambda^{3}\right),$$

where sum is taken only over the positive Bohr frequencies.

The analogy with Equation (22.6) of ([18] Chapter 22) also suggests the following definition of non-equilibrium free energy in a given state ρ

(37) $${\tilde{F}}_{\rho }\equiv {\langle \tilde{H}\rangle }_{P}+\beta^{-1}{\langle \ln P\rho \rangle }_{P}=F+\beta^{-1}S(P\rho ||{\tilde{\rho }}_{\beta }),$$

where ${\langle \;\cdot \;\rangle }_{P}\equiv \mathrm{Tr}\left(P\rho \;\cdot \;\right)$ and S(ρ||σ) is relative entropy ([36], Chapter 7.1). The only difference from Equation (22.6) of ([18] Chapter 22) consists of the fact that we use averaged state Pρ instead of ρ, which is natural in our setup.

The exact free energy is defined as

(38) $$F_{\rho }\equiv \langle H\rangle +\beta^{-1}\langle \ln\rho \rangle =F+\beta^{-1}S(\rho ||\rho_{\beta }),$$

where 〈·〉≡Tr(ρ·), which leads to

(39) $$F_{\rho }={\tilde{F}}_{\rho }+\Delta F_{\rho },$$

where similarly to Equation (33), $\Delta F_{\rho }$ has a definite sign, namely

(40) $$\Delta F_{\rho }=\beta^{-1}\left(S(\rho |\right|\rho_{\beta })-S(P\rho ||P\rho_{\beta }))\geq 0$$

due to monotonicity of the relative entropy under the completely positive map P ([36], Theorem 7.6). Similarly to $\tilde{S}$ and $\tilde{U}$, ${\tilde{F}}_{\rho }$ can be interpreted as observable free energy and $\Delta F_{\rho }$ as free energy hidden from our observations. As $\Delta F_{\rho }\geq 0$, we are always further from equilibrium than we think based on our restricted measurement possibilities. For example, if our exact non-equilibrium state is ${\tilde{\rho }}_{\beta }$, then it is impossible to distinguish it from $\rho_{\beta }$. Thus, its observable free energy coincides with the equilibrium one

(41) $${\tilde{F}}_{{\tilde{\rho }}_{\beta }}=F+\beta^{-1}S({\tilde{\rho }}_{\beta }\left|\right|{\tilde{\rho }}_{\beta })=F,$$

but $\Delta F_{{\tilde{\rho }}_{\beta }}$ is positive as in the general case. Namely, by Equations (37) and (38), we have

(42) $$\Delta F_{{\tilde{\rho }}_{\beta }}={\langle H\rangle }_{∼}-{\langle \tilde{H}\rangle }_{∼}.$$

As ${\langle H\rangle }_{∼}=\mathrm{Tr}HP\rho =\mathrm{Tr}HPP\rho_{\beta }=\mathrm{Tr}P\left(H\right)P\rho_{\beta }=\mathrm{Tr}H_{\mathrm{RWA}}\rho_{\beta }={\langle H_{\mathrm{RWA}}\rangle }_{∼}$, then

(43) $$\Delta F_{{\tilde{\rho }}_{\beta }}={\langle H_{\mathrm{RWA}}-\tilde{H}\rangle }_{∼}.$$

This formula is useful for asymptotic expansion of $\Delta F_{{\tilde{\rho }}_{\beta }}$ as the first two terms of the expansion of $\tilde{H}$ cancel $H_{\mathrm{RWA}}$ and the first non-trivial contribution is of order of $\lambda^{2}$ as in Equation (35). Namely, we have

(44) $$\Delta F_{{\tilde{\rho }}_{\beta }}=-\lambda^{2}{\langle H^{\left(2\right)}\rangle }_{∼}+O\left(\lambda^{3}\right)=-\lambda^{2}{\langle H^{\left(2\right)}\rangle }_{0}+O\left(\lambda^{3}\right).$$

Moreover, it is possible to show (see Appendix D) that ${\langle \partial_{\beta }H^{\left(2\right)}\rangle }_{0}=\beta^{-1}{\langle H^{\left(2\right)}\rangle }_{0}$, so

(45) $$\Delta U=\Delta F_{{\tilde{\rho }}_{\beta }}+O\left(\lambda^{3}\right),\;\Delta S=\beta \Delta F_{{\tilde{\rho }}_{\beta }}+O\left(\lambda^{3}\right).$$

The analogy with the mean force Hamiltonian can be made more explicit if one notes that the mean force Hamiltonian is closely related to the projector $P^{\prime }={\mathrm{Tr}}_{B}\left(\;\cdot \;\right)⊗\rho_{B}$ which is usually used for derivation of Markovian master equations and their perturbative corrections ([1], Subsection 9.1.1).

(46) $$P^{\prime }\frac{e^{-\beta H}}{Z}=\frac{1}{Z}{\mathrm{Tr}}_{B}e^{-\beta H}⊗\frac{1}{Z_{B}}e^{-\beta H_{B}}=\frac{1}{Z_{\mathrm{mf}}}e^{-\beta H_{\mathrm{mf}}}⊗\frac{1}{Z_{B}}e^{-\beta H_{B}},$$

where $Z_{\mathrm{mf}}=Z/Z_{B}$ [19]. Thus, a stricter analog of our effective Hamiltonian should be $H_{\mathrm{mf}}+H_{B}$ with partition function *Z*. However, it seems that for operational meaning of the mean force Hamiltonian, the information about $H_{B}$ is also important, which makes this analog more natural. Nevertheless, importance of information about $H_{B}$ (not $H_{\mathrm{mf}}$ only) is still discussible [37,38].

From the mathematical point of view, both of these projectors are so-called conditional expectations [39,40,41,42]. They are correspondent to different choices of observable degrees of freedom. This suggests that the mean force Hamiltonian theory could be generalized to arbitrary conditional expectations, and for specific conditional expectation P, it is performed in this work. Thus, it is possible to say that the effective Gibbs state with such generalized projectors define different effective quantum equilibrium thermodynamics.

Let us also mention that similarly to mean force Hamiltonian theory, we assume in our work that the whole system (containing both the system and the reservoir in the mean force Hamiltonian case) is at the same temperature. However, there are possible generalizations of such a setup when the system interacts with two (or more) reservoirs at different temperatures [43]. In such a case, a natural analog of $P^{\prime }$ is a projector $P^{\prime \prime }={\mathrm{Tr}}_{B_{1},B_{2}}\left(\;\cdot \;\right)⊗\rho_{B_{1},\beta_{1}}⊗\rho_{B_{2},\beta_{2}}$, where $\rho_{B_{1},\beta_{1}}$ and $\rho_{B_{2},\beta_{2}}$ are states of the heat baths with inverse temperatures $\beta_{1}$ and $\beta_{2}$, respectively. The above equations assuming only one temperature, e.g., Equations (28) and (29), are not applicable in this case, but Equations (30)–(32), which are fundamental for our approach, still have their meaning. This suggests that it is possible to generalize the framework presented here to include such a multitemperature case, but it is not fully covered by the approach presented here as the scope of the current paper was focused on the one-temperature case. Nevertheless, we think that it is one of the most promising directions for future study.

## 4. Mean Force Hamiltonian for Effective Gibbs State

Let us now consider a compound system, consisting of two subsystems *A* and *B*. Let us consider subsystem *B* as “reservoir”. Let us assume that $H_{0}=H_{A}⊗I+I⊗H_{B}$. Then, it is possible to define a mean for the Hamiltonian ${\tilde{H}}_{\mathrm{mf}}$ for the effective Gibbs state by the following formula

(47) $${\tilde{\rho }}_{\mathrm{mf}}\equiv {\mathrm{Tr}}_{B}P\rho_{\beta }=\frac{1}{{\tilde{Z}}_{\mathrm{mf}}}e^{-\beta {\tilde{H}}_{\mathrm{mf}}},$$

where ${\tilde{Z}}_{\mathrm{mf}}=\tilde{Z}/Z_{B}$, $Z_{B}\equiv {\mathrm{Tr}}_{B}e^{-\beta H_{B}}$. Then, similarly to Proposition 1, it is possible to obtain the perturbative expansion in λ for ${\tilde{H}}_{\mathrm{mf}}$ (see Appendix E).

**Proposition** **3.** *The perturbative expansion of ${\tilde{H}}_{\mathrm{mf}}$ in λ has the form*

(48) $${\tilde{H}}_{\mathrm{mf}}=H_{A}-\beta^{-1}\sum_{n=1}^{\infty }\lambda^{n}\sum_{k_{0}+\cdots +k_{m}=n}\frac{{\left(-1\right)}^{m}}{m+1}{\langle M_{k_{0}}\left(\beta \right)\rangle }_{B}{\langle M_{k_{1}}\left(\beta \right)\rangle }_{B}\cdots {\langle M_{k_{m}}\left(\beta \right)\rangle }_{B},$$

*where ${\langle \;\cdot \;\rangle }_{B}\equiv {\mathrm{Tr}}_{B}\left(\;\cdot \;Z_{B}^{-1}e^{-\beta H_{B}}\right)$.*

Here, $M_{k}\left(\beta \right)$ can also be calculated by Proposition 2. The first terms of the expansion for ${\tilde{H}}_{\mathrm{mf}}$ have the form

(49) $${\tilde{H}}_{\mathrm{mf}}=H_{A}+\lambda {\langle D_{0}\rangle }_{B}-\lambda^{2}\frac{\beta }{2}\left(\sum_{\omega \neq 0}f\left(\beta \omega \right){\langle D_{\omega }D_{\omega }^{†}\rangle }_{B}+{\langle D_{0}^{2}\rangle }_{B}-{\langle D_{0}\rangle }_{B}^{2}\right)+O\left(\lambda^{3}\right).$$

This formula can be made even more explicit if one considers the decomposition of $D_{\omega }$ into sum of eigenoperators of $\left[H_{A},\;\cdot \;\right]$ and $\left[H_{B},\;\cdot \;\right]$, i.e., similarly to Equation (17) introducing $A_{\omega }$ and $B_{\omega }$ such that

(50) $$\left[H_{A},A_{\omega_{1}}\right]=-\omega_{1}A_{\omega_{1}},\;\left[H_{B},B_{\omega_{2}}\right]=-\omega_{2}B_{\omega_{2}},$$

where $\omega_{1}$ and $\omega_{2}$ run over all possible Bohr frequencies of the Hamiltonians $H_{A}$ and $H_{B}$, respectively. Then, expansion (49) takes the form (see Appendix F)

(51) $$\begin{array}{c} {\tilde{H}}_{\mathrm{mf}}=H_{S}+\lambda {\langle B_{0}\rangle }_{B}A_{0}-\lambda^{2}\frac{\beta }{2}(\sum_{\omega_{1}\neq 0}\left(\sum_{\omega }f\left(\beta \omega \right){\langle B_{\omega_{1}+\omega }B_{\omega_{1}+\omega }^{†}\rangle }_{B}\right)A_{\omega_{1}}^{†}A_{\omega_{1}}\\ +\left(\sum_{\omega }f\left(\beta \omega \right){\langle B_{\omega }B_{\omega }^{†}\rangle }_{B}-{\langle B_{0}\rangle }_{B}^{2}\right)A_{0}^{2})+O\left(\lambda^{3}\right), \end{array}$$

where it is assumed that f(0)=1.

## 5. Examples

In this section, we consider several examples, and the notations are chosen in such a way as to emphasize the similarity between them. We use these examples to illustrate our formulae, but let us remark that, at least for the first and second model, it is possible to calculate the effective Hamiltonian exactly without perturbation theory; however, it is not the aim of our work. For all these examples, we consider two cases: the off-resonance and the resonance one. In this section, only the results are presented, all the calculations are given separately in Appendix G.

### 5.1. Two Interacting Two-Level Systems

Let us consider the two interacting two-level systems [44,45] *a* and *b*

(52) $$H=\omega_{a}\sigma_{a}^{+}\sigma_{a}^{-}+\omega_{b}\sigma_{b}^{+}\sigma_{b}^{-}+\lambda \left(\sigma_{a}^{-}+\sigma_{a}^{+}\right)\left(g^{*}\sigma_{b}^{-}+g\sigma_{b}^{+}\right),$$

where $\omega_{a}>0$, $\omega_{b}>0$ and $\sigma_{i}^{\pm }$ are usual ladder operators for two-level systems i=a,b.

(1) Off-resonance case $\omega_{a}\neq \omega_{b}$.

(53) $$\begin{array}{cc} {\tilde{H}}_{\mathrm{off}-\mathrm{res}}= & \omega_{a}n_{a}+\omega_{b}n_{b}-\lambda^{2}\frac{\beta }{2}{\left|g\right|}^{2}(f\left(\beta \left(\omega_{a}-\omega_{b}\right)\right)\left(1-n_{a}\right)n_{b}\\ \; & +f\left(\beta \left(\omega_{a}+\omega_{b}\right)\right)\left(1-n_{a}\right)\left(1-n_{b}\right)+f\left(\beta \left(\omega_{b}-\omega_{a}\right)\right)n_{a}\left(1-n_{b}\right)\\ \; & +f\left(\beta \left(-\omega_{a}-\omega_{b}\right)\right)n_{a}n_{b})+O\left(\lambda^{3}\right), \end{array}$$

where $n_{i}\equiv \sigma_{i}^{+}\sigma_{i}^{-}$ are number operators for i=a,b. In the leading order, the information loss has the form

(54) $$\Delta S_{\mathrm{off}-\mathrm{res}}=\lambda^{2}\beta {\left|g\right|}^{2}\frac{\omega_{a}\tanh\frac{\beta \omega_{a}}{2}-\omega_{b}\tanh\frac{\beta \omega_{b}}{2}}{\omega_{a}^{2}-\omega_{b}^{2}}+O\left(\lambda^{3}\right).$$

(2) Resonance case $\omega_{b}=\omega_{a}+\lambda \delta \omega$.

(55) $$\begin{array}{cc} {\tilde{H}}_{\mathrm{res}}= & \omega_{a}n_{a}+\omega_{b}n_{b}+\lambda \left(g\sigma_{a}^{-}\sigma_{b}^{+}+g^{*}\sigma_{a}^{+}\sigma_{b}^{-}\right)\\ \; & -\lambda^{2}\frac{\beta }{2}{\left|g\right|}^{2}\left(f\left(2\beta \omega_{a}\right)\left(1-n_{a}\right)\left(1-n_{b}\right)+f\left(-2\beta \omega_{a}\right)n_{a}n_{b}\right)+O\left(\lambda^{3}\right). \end{array}$$

In the leading order, the information loss has the form

(56) $$\Delta S_{\mathrm{res}}=\lambda^{2}\beta {\left|g\right|}^{2}\frac{\tanh\frac{\omega_{a}\beta }{2}}{2\omega_{a}}+O\left(\lambda^{3}\right).$$

Let us remark that it does not coincide with the off-resonance case with $\omega_{b}\to \omega_{a}$. Namely, we have

(57) $$\Delta S_{\mathrm{off}-\mathrm{res}}{|}_{\omega_{b}\to \omega_{a}}=\Delta S_{\mathrm{res}}+\lambda^{2}{\left(\frac{\beta |g|}{2\cosh\frac{\beta \omega_{a}}{2}}\right)}^{2}+O\left(\lambda^{3}\right).$$

Thus, off-resonance averaging leads to larger information loss even in the “resonance” limit than resonance averaging.

### 5.2. Two Interacting Harmonic Oscillators

Let us consider the two interacting harmonic oscillators

(58) $$H=\omega_{a}a^{†}a+\omega_{b}b^{†}b+\lambda \left(a+a^{†}\right)\left(g^{*}b+gb^{†}\right),$$

where $\omega_{a}>0$, $\omega_{b}>0$ and $a,a^{†}$ and $b,b^{†}$ are oscillator (bosonic) ladder operators. Averaging with respect to fast oscillations needed for so-called quasi-stationary states was recently discussed in [46].

(1) Off-resonance case $\omega_{a}\neq \omega_{b}$.

(59) $$\begin{array}{cc} {\tilde{H}}_{\mathrm{off}-\mathrm{res}}= & \omega_{a}n_{a}+\omega_{b}n_{b}-\lambda^{2}\frac{\beta }{2}{\left|g\right|}^{2}(f\left(\beta \left(\omega_{a}+\omega_{b}\right)\right)\left(1+n_{a}\right)n_{b}\\ \; & +f\left(\beta \left(\omega_{a}+\omega_{b}\right)\right)\left(1+n_{a}\right)\left(1+n_{b}\right)+f\left(\beta \left(\omega_{b}-\omega_{a}\right)\right)n_{a}\left(1+n_{b}\right)\\ \; & +f\left(\beta \left(-\omega_{a}-\omega_{b}\right)\right)n_{a}n_{b})+O\left(\lambda^{3}\right), \end{array}$$

where $n_{a}\equiv a^{†}a$, $n_{b}\equiv b^{†}b$. In the leading order, the information loss has the form

(60) $$\Delta S_{\mathrm{off}-\mathrm{res}}=\lambda^{2}\beta {\left|g\right|}^{2}\frac{\omega_{a}\coth\frac{\beta \omega_{b}}{2}-\omega_{b}\coth\frac{\beta \omega_{a}}{2}}{\omega_{a}^{2}-\omega_{b}^{2}}+O\left(\lambda^{3}\right).$$

(2) Resonance case $\omega_{b}=\omega_{a}+\lambda \delta \omega$.

(61) $$\begin{array}{cc} {\tilde{H}}_{\mathrm{res}}= & \omega_{a}n_{a}+\omega_{b}n_{b}+\lambda \left(gab^{†}+g^{*}a^{†}b\right)\\ \; & -\lambda^{2}\frac{\beta }{2}{\left|g\right|}^{2}\left(f\left(2\beta \omega_{a}\right)\left(1+n_{a}\right)\left(1+n_{b}\right)+f\left(-2\beta \omega_{a}\right)n_{a}n_{b}\right)+O\left(\lambda^{3}\right). \end{array}$$

In the leading order, the information loss has the form

(62) $$\Delta S_{\mathrm{res}}=\lambda^{2}\beta {\left|g\right|}^{2}\frac{\coth\frac{\omega_{a}\beta }{2}}{2\omega_{a}}+O\left(\lambda^{3}\right).$$

**Figure 1:**
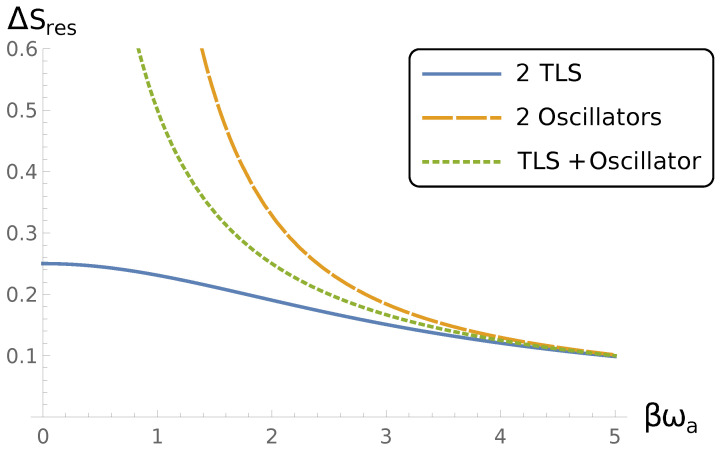
The information loss for resonance case and β|g|=1 for two two-level systems (solid line), two oscillators (dashed line) and two-level system interaction with oscillator (dotted line).

Interestingly, this quantity asymptotically coincides with Equation (56) for $\omega_{a}\beta \gg 1$ (see Figure 1). Similarly to Equation (57), we have

(63) $$\Delta S_{\mathrm{off}-\mathrm{res}}{|}_{\omega_{b}\to \omega_{a}}=\Delta S_{\mathrm{res}}+\lambda^{2}{\left(\frac{\beta |g|}{2\sinh\frac{\beta \omega_{a}}{2}}\right)}^{2}+O\left(\lambda^{3}\right).$$

### 5.3. Two-Level System Interacting with Harmonic Oscillator

Let us consider a two-level system interacting with a harmonic oscillator

(64) $$H=\omega_{a}\sigma^{+}\sigma^{-}+\omega_{b}b^{†}b+\lambda \left(\sigma^{+}+\sigma^{-}\right)\left(g^{*}b+gb^{†}\right),$$

where $\omega_{a}>0$, $\omega_{b}>0$ and $\sigma^{+},\sigma^{-}$ and $b,b^{†}$ are two-level and bosonic ladder operators, respectively.

(1) Off-resonance case $\omega_{a}\neq \omega_{b}$.

(65) $$\begin{array}{cc} {\tilde{H}}_{\mathrm{off}-\mathrm{res}}= & \omega_{a}n_{a}+\omega_{b}n_{b}-\lambda^{2}\frac{\beta }{2}{\left|g\right|}^{2}(f\left(\beta \left(\omega_{a}+\omega_{b}\right)\right)\left(1-n_{a}\right)n_{b}\\ \; & +f\left(\beta \left(\omega_{a}+\omega_{b}\right)\right)\left(1-n_{a}\right)\left(1+n_{b}\right)+f\left(\beta \left(\omega_{b}-\omega_{a}\right)\right)n_{a}\left(1+n_{b}\right)\\ \; & +f\left(\beta \left(-\omega_{a}-\omega_{b}\right)\right)n_{a}n_{b})+O\left(\lambda^{3}\right), \end{array}$$

where $n_{a}\equiv \sigma^{+}\sigma_{-}$, $n_{b}\equiv b^{†}b$. In the leading order, the information loss has the form

(66) $$\Delta S_{\mathrm{off}-\mathrm{res}}=\lambda^{2}\beta {\left|g\right|}^{2}\frac{\omega_{a}\tanh\frac{\beta \omega_{a}}{2}\coth\frac{\beta \omega_{b}}{2}-\omega_{b}}{\omega_{a}^{2}-\omega_{b}^{2}}+O\left(\lambda^{3}\right).$$

(2) Resonance case $\omega_{b}=\omega_{a}+\lambda \delta \omega$.

(67) $$\begin{array}{cc} {\tilde{H}}_{\mathrm{res}}= & \omega_{a}n_{a}+\omega_{b}n_{b}+\lambda \left(g\sigma^{-}b^{†}+g^{*}\sigma^{+}b\right)\\ \; & -\lambda^{2}\frac{\beta }{2}{\left|g\right|}^{2}\left(f\left(2\beta \omega_{a}\right)\left(1-n_{a}\right)\left(1+n_{b}\right)+f\left(-2\beta \omega_{a}\right)n_{a}n_{b}\right)+O\left(\lambda^{3}\right). \end{array}$$

In the leading order, the information loss has the form

(68) $$\Delta S_{\mathrm{res}}=\lambda^{2}\frac{{\beta |g|}^{2}}{2\omega_{a}}+O\left(\lambda^{3}\right).$$

This also asymptotically coincides with Equation (56) for $\omega_{a}\beta \gg 1$ (see Figure 1). Similarly to Equation (57), we have

(69) $$\Delta S_{\mathrm{off}-\mathrm{res}}{|}_{\omega_{b}\to \omega_{a}}=\Delta S_{\mathrm{res}}+\lambda^{2}\frac{{\beta |g|}^{2}}{2\sinh\beta \omega_{a}}+O\left(\lambda^{3}\right).$$

## 6. Conclusions

We have developed a systematic perturbative calculation of the effective Hamiltonian which defines the effective Gibbs state for the averaged observables. We have shown that the first two terms of the perturbative expansion of such an effective Hamiltonian coincide with the RWA Hamiltonian, and the second-order term of the expansion is the first non-trivial temperature-dependent term. It defines the leading order of the information loss due to the restricted observation capabilities in this setup and the leading order of the energy, which is not observable in our setup due to the same reason. We have shown the analogy between our setup and the mean force Hamiltonian. To deepen this analogy, we have also obtained the perturbative expansion for the mean force Hamiltonian for the effective Gibbs state. At the end, we have considered several examples, which illustrate the preceding material.

We think that the analogy between the mean force Hamiltonian and our effective Hamiltonians suggests the possibility to generalize our approach to form effective equilibrium quantum thermodynamics.

As it was already mentioned at the end of Section 3, a multitemperature generalization similar to [43] of the framework discussed in this work is a possible direction for further study. In particular, such a study could be important due to modern interest in such a multitemperature setup from the separability viewpoint [47].

## Data Availability

Not applicable.

## Abbreviations

The following abbreviations are used in this manuscript:

RWA	Rotating Wave Approximation

## Appendix A. Properties of Averaging Projector

Trace preservation of P follows from

(A1) $$\mathrm{Tr}PX=\lim_{T\to +\infty }\frac{1}{T}\int_{0}^{T}\mathrm{Tr}e^{iH_{0}t}Xe^{-iH_{0}t}dt=\lim_{T\to +\infty }\frac{1}{T}\int_{0}^{T}dt\mathrm{Tr}X=\mathrm{Tr}X,$$

Then, let us prove Property 3 first. For $H_{0}=\sum_{\varepsilon }\varepsilon \Pi_{\varepsilon }$, we have

(A2) $$PX=\lim_{T\to +\infty }\frac{1}{T}\int_{0}^{T}dte^{iH_{0}t}Xe^{-iH_{0}t}=\lim_{T\to +\infty }\frac{1}{T}\int_{0}^{T}dt\sum_{\varepsilon ,\varepsilon^{\prime }}e^{i(\varepsilon -\varepsilon^{\prime })t}\Pi_{\varepsilon }X\Pi_{\varepsilon^{\prime }}=\sum_{\varepsilon }\Pi_{\varepsilon }X\Pi_{\varepsilon }$$

because

(A3) $$\lim_{T\to +\infty }\frac{1}{T}\int_{0}^{T}dte^{i(\varepsilon -\varepsilon^{\prime })t}=\delta_{\varepsilon \varepsilon^{\prime }}.$$

As $\Pi_{\varepsilon }=\Pi_{\varepsilon }^{†}$, then they define Kraus representation [36], p. 110 of P, which proves Property 1. Calculating

(A4) $$P^{2}X=\sum_{\varepsilon ,\varepsilon^{\prime }}\Pi_{\varepsilon^{\prime }}\Pi_{\varepsilon }X\Pi_{\varepsilon }\Pi_{\varepsilon^{\prime }}=\sum_{\varepsilon ,\varepsilon^{\prime }}\delta_{\varepsilon \varepsilon^{\prime }}\Pi_{\varepsilon }X\Pi_{\varepsilon }=\sum_{\varepsilon }\Pi_{\varepsilon }X\Pi_{\varepsilon }=PX$$

and

(A5) $$\begin{array}{cc} \mathrm{Tr}X^{†}PY & =\mathrm{Tr}X^{†}\sum_{\varepsilon }\Pi_{\varepsilon }Y\Pi_{\varepsilon }\\ \; & =\sum_{\varepsilon }\mathrm{Tr}\Pi_{\varepsilon }X^{†}\Pi_{\varepsilon }Y=\mathrm{Tr}\sum_{\varepsilon }{\left(\Pi_{\varepsilon }X\Pi_{\varepsilon }\right)}^{†}Y=\mathrm{Tr}{\left(PX\right)}^{†}Y \end{array}$$

we obtain Property 2.

## Appendix B. Perturbative Expansion for Effective Hamiltonian

**Proof of Proposition 1.** Let us define $V\left(\beta \right)\equiv e^{\beta H_{0}}e^{-\beta H}$, then it satisfies

(A6) $$\frac{d}{d\beta }V\left(\beta \right)=-\lambda H_{I}\left(\beta \right)V\left(\beta \right),\;V\left(0\right)=I,$$

where $H_{I}\left(\beta \right)$ is defined by Equation (14). Namely,

(A7) $$\begin{array}{cc} \frac{d}{d\beta }\left(e^{\beta H_{0}}e^{-\beta H}\right) & =-e^{\beta H_{0}}He^{-\beta H}+e^{\beta H_{0}}H_{0}e^{-\beta H}\\ \; & =-e^{\beta H_{0}}\left(H-H_{0}\right)e^{-\beta H_{0}}\left(e^{\beta H_{0}}e^{-\beta H}\right)=-\lambda H_{I}\left(\beta \right)\left(e^{\beta H_{0}}e^{-\beta H}\right). \end{array}$$

Then, representing V(β) by the Dyson series and applying the projector P, one has

(A8) $$PV\left(\beta \right)=I+\sum_{k=1}^{\infty }\lambda^{k}M_{k}\left(\beta \right)$$

with $M_{k}\left(\beta \right)$ defined by Equation (13). By the Richter formula ([48], Equation (11.1)), one has

(A9) $$\log PV\left(\beta \right)=\int_{0}^{1}\left(PV\left(\beta \right)-I\right){\left(t\left(PV\left(\beta \right)-I\right)+I\right)}^{-1}dt$$

Then, we have

(A10) $${\left(t\left(PV\left(\beta \right)-I\right)+I\right)}^{-1}=\sum_{n=0}^{\infty }\lambda^{n}\sum_{k_{1}+\cdots +k_{m}=n}{\left(-1\right)}^{m}t^{m}M_{k_{1}}\left(\beta \right)\cdots M_{k_{m}}\left(\beta \right)$$

and

(A11) $$\begin{array}{cc} \left(PV(\beta )-I\right) & {\left(t\left(PV\left(\beta \right)-I\right)+I\right)}^{-1}\\ \; & =\sum_{n=1}\lambda^{n}\sum_{k_{0}+\cdots +k_{m}=n}{\left(-1\right)}^{m}t^{m}M_{k_{0}}\left(\beta \right)M_{k_{1}}\left(\beta \right)\cdots M_{k_{m}}\left(\beta \right) \end{array}$$

By substituting it in Equation (A9) and taking the integral, we have

(A12) $$\log PV\left(\beta \right)=\sum_{n=1}^{\infty }\lambda^{n}\sum_{k_{0}+\cdots +k_{m}=n}\frac{{\left(-1\right)}^{m}}{m+1}M_{k_{0}}\left(\beta \right)M_{k_{1}}\left(\beta \right)\cdots M_{k_{m}}\left(\beta \right).$$

Taking into account

(A13) $$PV\left(\beta \right)=Pe^{\beta H_{0}}e^{-\beta H}=\lim_{T\to +\infty }\frac{1}{T}\int_{0}^{T}e^{iH_{0}t}e^{\beta H_{0}}e^{-\beta H}e^{-iH_{0}t}dt=e^{\beta H_{0}}Pe^{-\beta H}$$

we have $Pe^{-\beta H}=e^{-\beta H_{0}}PV\left(\beta \right)$. Let us remark that $H_{0}$ commutes with any operator PX

(A14) $$\left[H_{0},PX\right]=\sum_{\varepsilon }\left(\varepsilon \Pi_{\varepsilon }X\Pi_{\varepsilon }-\Pi_{\varepsilon }X\Pi_{\varepsilon }\varepsilon \right)=0,$$

where Equation (11) was used. Thus, we have

(A15) $$Pe^{-\beta H}=e^{-\beta H_{0}}e^{\log PV(\beta )}=e^{-\beta (H_{0}-\beta^{-1}\log PV\left(\beta \right))}$$

and $\tilde{H}=H_{0}-\beta^{-1}\log PV\left(\beta \right)$, which along with Equation (A12) leads to Equation (12). □

## Appendix C. Eigenprojector Expansion

**Lemma** **A1.**
*The following formula holds*

(A16)
$$\begin{array}{cc} \int_{0}^{\beta }d\beta_{1} & \ldots \int_{0}^{\beta_{n-1}}d\beta_{n}e^{-\sum_{j=1}^{n}\beta_{j}\omega_{j}}\\ \; & =\frac{1}{\prod_{k=1}^{n}\sum_{j=1}^{k}\omega_{j}}+\sum_{p=1}^{n}{\left(-1\right)}^{p}\frac{e^{-\beta \sum_{i=1}^{p}\omega_{i}}}{\left(\prod_{m=1}^{p}\sum_{i=m}^{p}\omega_{i}\right)\left(\prod_{k=p+1}^{n}\sum_{j=p+1}^{k}\omega_{j}\right)}. \end{array}$$

**Proof.** Let us denote

(A17) $$h_{n}\left(\beta ;\omega_{1},\ldots ,\omega_{n}\right)=\int_{0}^{\beta }d\beta_{1}\ldots \int_{0}^{\beta_{n-1}}d\beta_{n}e^{-\sum_{j=1}^{n}\beta_{j}\omega_{j}},$$

then, by direct computation, we have

(A18) $$\begin{array}{c} h_{n+1}\left(\beta ;\omega_{1},\ldots ,\omega_{n+1}\right)=\int_{0}^{\beta }d\beta_{1}e^{-\omega_{1}\beta_{1}}h_{n}\left(\beta ;\omega_{2},\ldots ,\omega_{n+1}\right)=\frac{1}{\prod_{k=1}^{n}\sum_{j=1}^{k}\omega_{j+1}}\int_{0}^{\beta }e^{-\omega_{1}\beta_{1}}d\beta_{1}\\ +\sum_{p=1}^{n}{\left(-1\right)}^{p}\frac{1}{\left(\prod_{m=1}^{p}\sum_{i=m}^{p}\omega_{i+1}\right)\left(\prod_{k=p+1}^{n}\sum_{j=p+1}^{k}\omega_{j+1}\right)}\int_{0}^{\beta }e^{-\omega_{1}\beta_{1}}e^{-\beta_{1}\sum_{i=1}^{p}\omega_{i+1}}d\beta_{1}\\ =\frac{1}{\prod_{k=2}^{n+1}\sum_{j=2}^{k}\omega_{j}}\frac{1-e^{-\omega_{1}\beta_{1}}}{\omega_{1}}+\sum_{p=1}^{n}{\left(-1\right)}^{p}\frac{1}{\left(\prod_{m=2}^{p+1}\sum_{i=m}^{p+1}\omega_{i}\right)\left(\prod_{k=p+2}^{n+1}\sum_{j=p+2}^{k}\omega_{j}\right)}\frac{1-e^{-\beta \sum_{i=1}^{p+1}\omega_{i}}}{\sum_{i=1}^{p+1}\omega_{i}}\\ =\frac{1}{\prod_{k=2}^{n+1}\sum_{j=2}^{k}\omega_{j}}\frac{1-e^{-\omega_{1}\beta_{1}}}{\omega_{1}}-\sum_{p=2}^{n+1}{\left(-1\right)}^{p}\frac{1}{\left(\prod_{m=2}^{p}\sum_{i=m}^{p}\omega_{i}\right)\left(\prod_{k=p+1}^{n+1}\sum_{j=p+1}^{k}\omega_{j}\right)}\frac{1-e^{-\beta \sum_{i=1}^{p}\omega_{i}}}{\sum_{i=1}^{p}\omega_{i}}\\ =\frac{1}{\prod_{k=2}^{n+1}\sum_{j=2}^{k}\omega_{j}}\frac{1}{\omega_{1}}-\sum_{p=2}^{n+1}{\left(-1\right)}^{p}\frac{1}{\left(\prod_{m=1}^{p}\sum_{i=m}^{p}\omega_{i}\right)\left(\prod_{k=p+1}^{n+1}\sum_{j=p+1}^{k}\omega_{j}\right)}\\ -\frac{1}{\prod_{k=2}^{n+1}\sum_{j=2}^{k}\omega_{j}}\frac{e^{-\beta \omega_{1}}}{\omega_{1}}+\sum_{p=2}^{n+1}{\left(-1\right)}^{p}\frac{1}{\left(\prod_{m=1}^{p}\sum_{i=m}^{p}\omega_{i}\right)\left(\prod_{k=p+1}^{n+1}\sum_{j=p+1}^{k}\omega_{j}\right)}e^{-\beta \sum_{i=1}^{p}\omega_{i}}\\ =-\sum_{p=1}^{n+1}{\left(-1\right)}^{p}\frac{1}{\left(\prod_{m=1}^{p}\sum_{i=m}^{p}\omega_{i}\right)\left(\prod_{k=p+1}^{n+1}\sum_{j=p+1}^{k}\omega_{j}\right)}\\ +\sum_{p=1}^{n+1}{\left(-1\right)}^{p}\frac{1}{\left(\prod_{m=1}^{p}\sum_{i=m}^{p}\omega_{i}\right)\left(\prod_{k=p+1}^{n+1}\sum_{j=p+1}^{k}\omega_{j}\right)}e^{-\beta \sum_{i=1}^{p}\omega_{i}}. \end{array}$$

Using

(A19) $$h_{n}\left(0;\omega_{1},\ldots ,\omega_{n}\right)=0$$

we have

(A20) $$\sum_{p=1}^{n}{\left(-1\right)}^{p}\frac{1}{\left(\prod_{m=1}^{p}\sum_{i=m}^{p}\omega_{i}\right)\left(\prod_{k=p+1}^{n}\sum_{j=p+1}^{k}\omega_{j}\right)}=-\frac{1}{\prod_{k=1}^{n}\sum_{j=1}^{k}\omega_{j}},$$

then

(A21) $$\begin{array}{cc} \; & \sum_{p=1}^{n+1}{\left(-1\right)}^{p}\frac{1}{\left(\prod_{m=1}^{p}\sum_{i=m}^{p}\omega_{i}\right)\left(\prod_{k=p+1}^{n+1}\sum_{j=p+1}^{k}\omega_{j}\right)}\\ \; & =\sum_{p=1}^{n}{\left(-1\right)}^{p}\frac{1}{\left(\prod_{m=1}^{p}\sum_{i=m}^{p}\omega_{i}\right)\left(\prod_{k=p+1}^{n}\sum_{j=p+1}^{k}\omega_{j}\right)}\frac{1}{\sum_{j=n+1}^{n+1}\omega_{j}}+{\left(-1\right)}^{n+1}\frac{1}{\left(\prod_{m=1}^{n+1}\sum_{i=m}^{n+1}\omega_{i}\right)}\\ \; & =-\frac{1}{\prod_{k=1}^{n}\sum_{j=1}^{k}\omega_{j}}\frac{1}{\omega_{n+1}}+{\left(-1\right)}^{n+1}\frac{1}{\prod_{m=1}^{n}\left(\sum_{i=m}^{n}\omega_{i}+\omega_{n+1}\right)}\frac{1}{\omega_{n+1}}=\frac{1}{\prod_{k=1}^{n+1}\sum_{j=1}^{k}\omega_{j}}. \end{array}$$

Substituting it in Equation (A18), we obtain Equation (A16). □

**Lemma** **A2.**
*The following formula holds*

(A22)
$$\begin{array}{c} \int_{0}^{\beta }d\beta_{1}\ldots \int_{0}^{\beta_{n-1}}d\beta_{n}e^{-\sum_{j=1}^{n-1}\beta_{j}\omega_{j}+\beta_{n}\sum_{j=1}^{n-1}\omega_{j}}=\frac{1}{\prod_{k=1}^{n-1}\sum_{j=1}^{k}\omega_{j}}\left(\beta -\sum_{k=1}^{n-1}\frac{1}{\sum_{j=1}^{k}\omega_{j}}\right)\\ -\sum_{p=1}^{n-1}\frac{{\left(-1\right)}^{p}}{\left(\prod_{m=2}^{p}\sum_{i=m}^{p}\omega_{i}\right){\left(\sum_{r=1}^{p}\omega_{r}\right)}^{2}\left(\prod_{k=p+1}^{n-1}\sum_{j=p+1}^{k}\omega_{j}\right)}e^{-\beta \sum_{i=1}^{p}\omega_{i}}. \end{array}$$

**Proof.** From Lemma A1, we have

(A23) $$\begin{array}{cc} \; & h_{n}\left(\beta ;\omega_{1},\ldots ,\omega_{n}\right)=\frac{1}{\sum_{j=1}^{n}\omega_{j}\prod_{k=1}^{n-1}\sum_{j=1}^{k}\omega_{j}}\\ \; & +\sum_{p=1}^{n-1}{\left(-1\right)}^{p}\frac{e^{-\beta \sum_{i=1}^{p}\omega_{i}}}{\left(\prod_{m=1}^{p}\sum_{i=m}^{p}\omega_{i}\right)\left(\prod_{k=p+1}^{n}\sum_{j=p+1}^{k}\omega_{j}\right)}+{\left(-1\right)}^{n}\frac{e^{-\beta \sum_{i=1}^{n}\omega_{i}}}{\sum_{i=1}^{n}\omega_{i}\left(\prod_{m=2}^{n}\sum_{i=m}^{n}\omega_{i}\right)}. \end{array}$$

Taking the limit

(A24) $$\sum_{i=1}^{n}\omega_{i}\to 0$$

we obtain Equation (A22). □

**Proof of Proposition 2.** Using expansion (16) and (17), we have

(A25) $$H_{I}\left(\beta \right)=e^{\beta H_{0}}H_{I}e^{-\beta H_{0}}=\sum_{\omega }e^{-\beta \omega }D_{\omega }.$$

Then

(A26) $$PH_{I}\left(\beta_{1}\right)\cdots H_{I}\left(\beta_{k}\right)=\sum_{\omega_{1},\ldots ,\omega_{k}}e^{-\beta_{1}\omega_{1}-\ldots -\beta_{k}\omega_{k}}P\left(D_{\omega_{1}}\ldots D_{\omega_{k}}\right).$$

Let us calculate

(A27) $$\begin{array}{cc} P(D_{\omega_{1}}\ldots D_{\omega_{k}}) & =\lim_{T\to +\infty }\frac{1}{T}\int_{0}^{T}dte^{iH_{0}t}D_{\omega_{1}}\ldots D_{\omega_{k}}e^{-iH_{0}t}\\ \; & =\lim_{T\to +\infty }\frac{1}{T}\int_{0}^{T}dt\;e^{iH_{0}t}D_{\omega_{1}}e^{-iH_{0}t}\ldots e^{iH_{0}t}D_{\omega_{k}}e^{-iH_{0}t}\\ \; & =\lim_{T\to +\infty }\frac{1}{T}\int_{0}^{T}dt\;e^{i(\omega_{1}+\cdots +\omega_{k})t}D_{\omega_{1}}\cdots D_{\omega_{k}}\\ \; & =D_{\omega_{1}}\cdots D_{\omega_{k-1}}D_{-\omega_{1}-\ldots -\omega_{k-1}}\delta_{\omega_{1}+\cdot +\omega_{k},0}. \end{array}$$

Substituting it in Equation (A26), we have

(A28) $$\begin{array}{cc} \; & PH_{I}\left(\beta_{1}\right)\cdots H_{I}\left(\beta_{k}\right)\\ \; & =\sum_{\omega_{1},\ldots ,\omega_{k-1}}e^{-\left(\beta_{1}-\beta_{k}\right)\omega_{1}-\ldots -\left(\beta_{k-1}-\beta_{k}\right)\omega_{k-1}}D_{\omega_{1}}\cdots D_{\omega_{k-1}}D_{-\omega_{1}-\ldots -\omega_{k-1}}. \end{array}$$

Then, by Equation (13) and Lemma A2, we have Equation (18). □

Several first operators $M_{k}\left(\beta \right)$ are

(A29) $$\begin{array}{cc} M_{1}\left(\beta \right) & =-\beta D_{0}, \end{array}$$

(A30) $$\begin{array}{cc} M_{2}\left(\beta \right) & =\sum_{\omega }\frac{\beta \omega +e^{-\beta \omega }-1}{\omega^{2}}D_{\omega }D_{-\omega }=\sum_{\omega \neq 0}\frac{\beta \omega +e^{-\beta \omega }-1}{\omega^{2}}D_{\omega }D_{\omega }^{†}+\frac{\beta }{2}D_{0}^{2}, \end{array}$$

(A31) $$\begin{array}{cc} M_{3}\left(\beta \right) & =-\sum_{\omega_{1},\omega_{2}}\left(\frac{\beta -\frac{1}{\omega_{1}}-\frac{1}{\omega_{1}+\omega_{2}}}{\omega_{1}\left(\omega_{1}+\omega_{2}\right)}+\frac{e^{-\beta \omega_{1}}}{\omega_{1}^{2}\omega_{2}}-\frac{e^{-\beta \left(\omega_{1}+\omega_{2}\right)}}{\omega_{2}{\left(\omega_{1}+\omega_{2}\right)}^{2}}\right)D_{\omega_{1}}D_{\omega_{2}}D_{-\omega_{1}-\omega_{2}}. \end{array}$$

This leads to

(A32) $$M_{2}\left(\beta \right)-\frac{1}{2}{\left(M_{1}\left(\beta \right)\right)}^{2}=\sum_{\omega \neq 0}\frac{\beta \omega +e^{-\beta \omega }-1}{\omega^{2}}D_{\omega }D_{\omega }^{†}.$$

Substituting this expression and $M_{1}\left(\beta \right)$ in Equation (15) leads to Equation (20).

Similarly, higher-order cumulants could be calculated, e.g.,

(A33) $$\begin{array}{cc} \; & M_{3}\left(\beta \right)-\frac{1}{2}M_{2}\left(\beta \right)M_{1}\left(\beta \right)-\frac{1}{2}M_{1}\left(\beta \right)M_{2}\left(\beta \right)+\frac{1}{3}{\left(M_{1}\left(\beta \right)\right)}^{3}\\ \; & =\sum_{\omega_{1},\omega_{2}}\left(\frac{\beta -\frac{1}{\omega_{1}}-\frac{1}{\omega_{1}+\omega_{2}}}{\omega_{1}\left(\omega_{1}+\omega_{2}\right)}+\frac{e^{-\beta \omega_{1}}}{\omega_{1}^{2}\omega_{2}}-\frac{e^{-\beta \left(\omega_{1}+\omega_{2}\right)}}{\omega_{2}{\left(\omega_{1}+\omega_{2}\right)}^{2}}\right)D_{\omega_{1}}D_{\omega_{2}}D_{-\omega_{1}-\omega_{2}}\\ \; & +\beta \frac{1}{2}\sum_{\omega }\frac{\beta \omega +e^{-\beta \omega }-1}{\omega^{2}}D_{\omega }D_{-\omega }D_{0}+\beta \frac{1}{2}\sum_{\omega }\frac{\beta \omega +e^{-\beta \omega }-1}{\omega^{2}}D_{0}D_{\omega }D_{-\omega }-\frac{1}{3}\beta^{3}D_{0}^{3}. \end{array}$$

## Appendix D. Average of Second Correction with Respect to Gibbs State for Free Hamiltonian

Let us express ${\langle D_{\omega }^{†}D_{\omega }\rangle }_{0}$ in terms of ${\langle D_{\omega }D_{\omega }^{†}\rangle }_{0}$ as

(A34) $$\begin{array}{cc} \; & {\langle D_{\omega }^{†}D_{\omega }\rangle }_{0}=\mathrm{Tr}D_{\omega }^{†}D_{\omega }Z^{-1}e^{-\beta H_{0}}=Z^{-1}\mathrm{Tr}D_{\omega }^{†}e^{-\beta H_{0}}e^{\beta H_{0}}D_{\omega }e^{-\beta H_{0}}\\ \; & =Z^{-1}\mathrm{Tr}D_{\omega }^{†}e^{-\beta H_{0}}e^{-\beta \omega }D_{\omega }=e^{-\beta \omega }\mathrm{Tr}D_{\omega }D_{\omega }^{†}Z^{-1}e^{-\beta H_{0}}=e^{-\beta \omega }{\langle D_{\omega }D_{\omega }^{†}\rangle }_{0}. \end{array}$$

Taking into account Equation (23), we have

(A35) $$\begin{array}{cc} -{\langle {\tilde{H}}^{\left(2\right)}\rangle }_{0} & =\frac{\beta }{2}\sum_{\omega \neq 0}f\left(\beta \omega \right){\langle D_{\omega }D_{\omega }^{†}\rangle }_{0}=\frac{\beta }{2}\sum_{\omega >0}\left(f\left(\beta \omega \right){\langle D_{\omega }D_{\omega }^{†}\rangle }_{0}+f\left(-\beta \omega \right){\langle D_{\omega }^{†}D_{\omega }\rangle }_{0}\right)\\ \; & =\beta \sum_{\omega >0}\frac{1}{2}\left(f\left(\beta \omega \right)+e^{-\beta \omega }f\left(-\beta \omega \right)\right)\langle D_{\omega }D_{\omega }^{†}=\sum_{\omega >0}\frac{1-e^{-\beta \omega }}{\omega }{\langle D_{\omega }D_{\omega }^{†}\rangle }_{0}. \end{array}$$

Similarly, taking into account Equation (25), we have

(A36) $$\begin{array}{cc} -{\langle \partial_{\beta }{\tilde{H}}^{\left(2\right)}\rangle }_{0}=\frac{\beta }{2}\sum_{\omega \neq 0}f_{1}\left(\beta \omega \right){\langle D_{\omega }D_{\omega }^{†}\rangle }_{0}\; & =\sum_{\omega >0}\frac{1}{2}\left(f_{1}\left(\beta \omega \right)+e^{-\beta \omega }f_{1}\left(-\beta \omega \right)\right){\langle D_{\omega }D_{\omega }^{†}\rangle }_{0}\\ \; & =\sum_{\omega >0}\frac{1-e^{-\beta \omega }}{\beta \omega }{\langle D_{\omega }D_{\omega }^{†}\rangle }_{0}=\frac{1}{\beta }{\langle {\tilde{H}}^{\left(2\right)}\rangle }_{0}. \end{array}$$

## Appendix E. Perturbative Expansion of Mean Force Hamiltonian for Effective Gibbs State

**Proof of Proposition 3.** Taking into account Equation (A13), we have

(A37) $$\begin{array}{cc} {\mathrm{Tr}}_{B}Pe^{-\beta H} & ={\mathrm{Tr}}_{B}e^{-\beta H_{0}}PV\left(\beta \right)\\ \; & =e^{-\beta H_{S}}{\mathrm{Tr}}_{B}e^{-\beta H_{B}}PV\left(\beta \right)=e^{-\beta H_{S}}{\mathrm{Tr}}_{B}PV\left(\beta \right)e^{-\beta H_{B}}. \end{array}$$

Due to Equation (A14), it can also be written as

(A38) $${\mathrm{Tr}}_{B}Pe^{-\beta H}={\mathrm{Tr}}_{B}PV\left(\beta \right)e^{-\beta H_{0}}=\left({\mathrm{Tr}}_{B}PV\left(\beta \right)e^{-\beta H_{B}}\right)e^{-\beta H_{S}},$$

so

(A39) $$[{\mathrm{Tr}}_{B}PV\left(\beta \right)e^{-\beta H_{B}},e^{-\beta H_{S}}]=0.$$

By Equation (A8), we have

(A40) $$\begin{array}{cc} {\mathrm{Tr}}_{B}PV\left(\beta \right)e^{-\beta H_{B}} & ={\mathrm{Tr}}_{B}e^{-\beta H_{B}}\left(\sum_{k=0}^{\infty }\lambda^{k}PM_{k}\left(\beta \right)\right)\\ \; & =Z_{B}\left(1+\sum_{k=1}^{\infty }\lambda^{k}{\mathrm{Tr}}_{B}Z_{B}^{-1}e^{-\beta H_{B}}M_{k}\left(\beta \right)\right)=Z_{B}\sum_{k=1}^{\infty }\lambda^{k}{\langle M_{k}\left(\beta \right)\rangle }_{B}. \end{array}$$

Taking into account Equation (A37), we have

(A41) $$Z_{B}^{-1}e^{-\beta {\tilde{H}}_{\mathrm{mf}}}=Z_{B}^{-1}{\mathrm{Tr}}_{B}Pe^{-\beta H}=e^{-\beta H_{S}}\sum_{k=1}^{\infty }\lambda^{k}{\langle M_{k}\left(\beta \right)\rangle }_{B}.$$

Then, the proof follows the proof of Proposition 1 (see Appendix B), replacing $M_{k}\left(\beta \right)$ with ${\langle M_{k}\left(\beta \right)\rangle }_{B}$ and $H_{0}$ with $H_{S}$. □

Similarly to Equation (15), the first several terms are

(A42) $${\tilde{H}}_{\mathrm{mf}}=H_{S}-\lambda \beta^{-1}{\langle M_{1}\left(\beta \right)\rangle }_{B}-\beta^{-1}\lambda^{2}\left({\langle M_{2}\left(\beta \right)\rangle }_{B}-\frac{1}{2}{\left({\langle M_{1}\left(\beta \right)\rangle }_{B}\right)}^{2}\right)+O\left(\lambda^{3}\right)$$

or using Proposition 2, similarly to Equation (20), we have Equation (49).

## Appendix F. Calculation of Mean Force Hamiltonian

Due to Equation (50), we have

(A43) $$D_{\omega }=\sum_{\omega_{1}}A_{\omega -\omega_{1}}⊗B_{\omega_{1}}=\sum_{\omega_{1}}A_{\omega_{1}-\omega }^{†}⊗B_{\omega_{1}}=\sum_{\omega_{1}}A_{\omega_{1}}^{†}⊗B_{\omega_{1}+\omega },$$

then

(A44) $$D_{\omega }D_{\omega }^{†}=\sum_{\omega_{1},\omega_{2}}A_{\omega_{1}}^{†}A_{\omega_{2}}⊗B_{\omega_{1}+\omega }B_{\omega_{2}+\omega }^{†}.$$

The second equation of Equation (50) also leads to $e^{\beta H_{B}}B_{\omega }e^{-\beta H_{B}}=e^{\beta [H_{B},\;\cdot \;]}B_{\omega }=e^{-\beta \omega }B_{\omega }$, then $B_{\omega }e^{-\beta H_{B}}=e^{-\beta \omega }e^{-\beta H_{B}}B_{\omega }$. Applying trace to both sides of this equation, we have $\left(1-e^{-\beta \omega }\right){\mathrm{Tr}}_{B}B_{\omega }e^{-\beta H_{B}}=0$. Thus, we have

(A45) $$\begin{array}{cc} {\langle B_{\omega }\rangle }_{B} & ={\langle B_{0}\rangle }_{B}\delta_{\omega ,0},\\ {\langle B_{\omega_{1}}B_{\omega_{2}}^{†}\rangle }_{B} & ={\langle B_{\omega_{1}}B_{\omega_{1}}^{†}\rangle }_{B}\delta_{\omega_{1},\omega_{2}}. \end{array}$$

Then, Equations (A43) and (A44) take the form

(A46) $${\langle D_{0}\rangle }_{B}=\sum_{\omega_{1}}A_{-\omega_{1}}{\langle B_{\omega_{1}}\rangle }_{B}=A_{0}{\langle B_{0}\rangle }_{B}$$

and

(A47) $$\begin{array}{c} {\langle D_{\omega }D_{\omega }^{†}\rangle }_{B}=\sum_{\omega_{1},\omega_{2}}A_{\omega_{1}}^{†}A_{\omega_{2}}{\langle B_{\omega_{1}+\omega }B_{\omega_{2}+\omega }^{†}\rangle }_{B}=\sum_{\omega_{1}}A_{\omega_{1}}^{†}A_{\omega_{1}}{\langle B_{\omega_{1}+\omega }B_{\omega_{1}+\omega }^{†}\rangle }_{B}\\ =\sum_{\omega_{1}\neq 0}A_{\omega_{1}}^{†}A_{\omega_{1}}{\langle B_{\omega_{1}+\omega }B_{\omega_{1}+\omega }^{†}\rangle }_{B}+A_{0}^{2}{\langle B_{\omega }B_{\omega }^{†}\rangle }_{B}. \end{array}$$

Hence, after substituting these formulae into Equation (49), we have

(A48) $$\begin{array}{cc} {\tilde{H}}_{\mathrm{mf}}=H_{S}+\lambda {\langle B_{0}\rangle }_{B}A_{0}\; & -\lambda^{2}\frac{\beta }{2}(\left(\sum_{\omega \neq 0,\omega_{1}\neq 0}f\left(\beta \omega \right){\langle B_{\omega_{1}+\omega }B_{\omega_{1}+\omega }^{†}\rangle }_{B}+{\langle B_{\omega_{1}}B_{\omega_{1}}^{†}\rangle }_{B}\right)A_{\omega_{1}}^{†}A_{\omega_{1}}\\ \; & +A_{0}^{2}\left(\sum_{\omega \neq 0}f\left(\beta \omega \right){\langle B_{\omega }B_{\omega }^{†}\rangle }_{B}+\langle B_{0}^{2}\rangle -{\langle B_{0}\rangle }_{B}^{2}\right))+O\left(\lambda^{3}\right). \end{array}$$

Assuming by continuity f(0)=1, this equation reduces to Equation (51).

## Appendix G. Calculations for the Examples

We provide fewer details for the second and third examples because they are fully analogous to the first one.

### Appendix G.1. Two Two-Level Systems

(1) For the off-resonance case, we have

(A49) $$H_{0}=\omega_{a}\sigma_{a}^{+}\sigma_{a}^{-}+\omega_{b}\sigma_{b}^{+}\sigma_{b}^{-},\;H_{I}=\left(\sigma_{a}^{-}+\sigma_{a}^{+}\right)\left(g^{*}\sigma_{b}^{-}+g\sigma_{b}^{+}\right).$$

As $\left[\omega_{i}\sigma_{i}^{+}\sigma_{i}^{-},\sigma_{i}^{\pm }\right]=-\left(\mp \omega_{i}\right)\sigma_{i}^{\pm }$ for i=a,b, then

(A50) $$D_{\omega_{a}-\omega_{b}}=D_{\omega_{b}-\omega_{a}}^{†}=g\sigma_{a}^{-}\sigma_{b}^{+},\;D_{\omega_{a}+\omega_{b}}=D_{-(\omega_{a}+\omega_{b})}^{†}=g^{*}\sigma_{a}^{-}\sigma_{b}^{-}.$$

As $n_{i}=\sigma_{i}^{+}\sigma_{i}^{-}=1-\sigma_{i}^{-}\sigma_{i}^{+}$ for i=a,b, then

(A51) $$\begin{array}{cc} D_{\omega_{a}-\omega_{b}}D_{\omega_{a}-\omega_{b}}^{†} & ={\left|g\right|}^{2}\sigma_{a}^{-}\sigma_{a}^{+}\sigma_{b}^{+}\sigma_{b}^{-}={\left|g\right|}^{2}\left(1-n_{a}\right)n_{b}, \end{array}$$

(A52) $$\begin{array}{cc} D_{\omega_{a}-\omega_{b}}^{†}D_{\omega_{a}-\omega_{b}} & ={\left|g\right|}^{2}\sigma_{a}^{+}\sigma_{a}^{-}\sigma_{b}^{-}\sigma_{b}^{+}={\left|g\right|}^{2}n_{a}\left(1-n_{b}\right), \end{array}$$

(A53) $$\begin{array}{cc} D_{\omega_{a}+\omega_{b}}D_{\omega_{a}+\omega_{b}}^{†} & ={\left|g\right|}^{2}\sigma_{a}^{-}\sigma_{a}^{+}\sigma_{b}^{-}\sigma_{b}^{+}={\left|g\right|}^{2}\left(1-n_{a}\right)\left(1-n_{b}\right), \end{array}$$

(A54) $$\begin{array}{cc} D_{-\omega_{a}-\omega_{b}}D_{-\omega_{a}-\omega_{b}}^{†} & ={\left|g\right|}^{2}\sigma_{a}^{+}\sigma_{a}^{-}\sigma_{b}^{+}\sigma_{b}^{-}={\left|g\right|}^{2}n_{a}n_{b}. \end{array}$$

Substituting it in Equation (20), we obtain Equation (53).

As

(A55) $${\langle n_{i}\rangle }_{0}\equiv \frac{\mathrm{Tr}n_{i}e^{-\beta \omega_{i}n_{i}}}{\mathrm{Tr}e^{-\beta \omega_{i}n_{i}}}=\frac{1}{e^{\beta \omega_{i}}+1}$$

for i=a,b, then by Equation (A35), we have

(A56) $$\begin{array}{cc} -{\langle H^{\left(2\right)}\rangle }_{0} & ={\left|g\right|}^{2}\left(\frac{1-e^{-\beta (\omega_{a}-\omega_{b})}}{\omega_{a}-\omega_{b}}\left(1-{\langle n_{a}\rangle }_{0}\right){\langle n_{b}\rangle }_{0}+\frac{1-e^{-\beta (\omega_{a}+\omega_{b})}}{\omega_{a}+\omega_{b}}\left(1-{\langle n_{a}\rangle }_{0}\right)\left(1-{\langle n_{b}\rangle }_{0}\right)\right)\\ \; & ={\left|g\right|}^{2}\frac{\omega_{a}\tanh\frac{\beta \omega_{a}}{2}-\omega_{b}\tanh\frac{\beta \omega_{b}}{2}}{\omega_{a}^{2}-\omega_{b}^{2}}. \end{array}$$

Thus, by Equation (36), we obtain Equation (54).

(2) For the resonance case, we have

(A57) $$H_{0}=\omega_{a}\left(\sigma_{a}^{+}\sigma_{a}^{-}+\sigma_{b}^{+}\sigma_{b}^{-}\right),\;H_{I}=\left(\sigma_{a}^{-}+\sigma_{a}^{+}\right)\left(g^{*}\sigma_{b}^{-}+g\sigma_{b}^{+}\right)+\delta \omega \sigma_{b}^{+}\sigma_{b}^{-}.$$

Now, the terms analogous to $D_{\omega_{a}-\omega_{b}}$ and $D_{\omega_{b}-\omega_{a}}$ contribute to $D_{0}$

(A58) $$D_{0}=D_{0}^{†}=\left(g\sigma_{a}^{-}\sigma_{b}^{+}+g^{*}\sigma_{a}^{+}\sigma_{b}^{-}\right)+\delta \omega \sigma_{b}^{+}\sigma_{b}^{-},\;D_{\omega_{a}+\omega_{b}}=D_{-(\omega_{a}+\omega_{b})}^{†}=g^{*}\sigma_{a}^{-}\sigma_{b}^{-}.$$

Substituting it in Equation (20), we obtain Equation (55).

As

(A59) $${\langle n_{i}\rangle }_{0}=\frac{1}{e^{\beta \omega_{a}}+1},$$

we have

(A60) $$-{\langle H^{\left(2\right)}\rangle }_{0}={\left|g\right|}^{2}\frac{1-e^{-2\beta \omega_{a}}}{2\omega_{a}}\left(1-{\langle n_{a}\rangle }_{0}\right)\left(1-{\langle n_{b}\rangle }_{0}\right)={\left|g\right|}^{2}\frac{\tanh\frac{\omega_{a}\beta }{2}}{2\omega_{a}}.$$

Thus, by Equation (36), we obtain Equation (56).

### Appendix G.2. Two Oscillators

(1) For the off-resonance case, we have

(A61) $$H_{0}=\omega_{a}a^{†}a+\omega_{b}b^{†}b,\;H_{I}=\left(a+a^{†}\right)\left(g^{*}b+gb^{†}\right),$$

(A62) $$D_{\omega_{a}-\omega_{b}}=D_{\omega_{b}-\omega_{a}}^{†}=gab^{†},\;D_{\omega_{a}+\omega_{b}}=D_{-(\omega_{a}+\omega_{b})}^{†}=g^{*}ab$$

and

(A63) $$\begin{array}{cc} D_{\omega_{a}-\omega_{b}}D_{\omega_{a}-\omega_{b}}^{†} & ={\left|g\right|}^{2}aa^{†}b^{†}b={\left|g\right|}^{2}\left(n_{a}+1\right)n_{b}, \end{array}$$

(A64) $$\begin{array}{cc} D_{\omega_{a}+\omega_{b}}D_{\omega_{a}+\omega_{b}}^{†} & ={\left|g\right|}^{2}aa^{†}bb^{†}={\left|g\right|}^{2}\left(n_{a}+1\right)\left(n_{b}+1\right), \end{array}$$

(A65) $$\begin{array}{cc} D_{\omega_{b}-\omega_{a}}D_{\omega_{b}-\omega_{a}}^{†} & ={\left|g\right|}^{2}a^{†}abb^{†}={\left|g\right|}^{2}n_{a}\left(n_{b}+1\right), \end{array}$$

(A66) $$\begin{array}{cc} D_{-(\omega_{a}+\omega_{b})}D_{-(\omega_{a}+\omega_{b})}^{†} & ={\left|g\right|}^{2}a^{†}ab^{†}b={\left|g\right|}^{2}n_{a}n_{b}. \end{array}$$

Substituting it in Equation (20), we obtain Equation (59).

As

(A67) $${\langle n_{i}\rangle }_{0}=\frac{1}{e^{\beta \omega_{i}}-1}$$

for i=a,b, then by Equation (A35), we have

(A68) $$\begin{array}{cc} -{\langle H^{\left(2\right)}\rangle }_{0} & ={\left|g\right|}^{2}\left(\frac{1-e^{-\beta (\omega_{a}-\omega_{b})}}{\omega_{a}-\omega_{b}}\left(1+{\langle n_{a}\rangle }_{0}\right){\langle n_{b}\rangle }_{0}+\frac{1-e^{-\beta (\omega_{a}+\omega_{b})}}{\omega_{a}+\omega_{b}}\left(1+{\langle n_{a}\rangle }_{0}\right)\left(1+{\langle n_{b}\rangle }_{0}\right)\right)\\ \; & ={\left|g\right|}^{2}\frac{\omega_{a}\coth\frac{\beta \omega_{b}}{2}-\omega_{b}\coth\frac{\beta \omega_{a}}{2}}{\omega_{a}^{2}-\omega_{b}^{2}}. \end{array}$$

By Equation (36), we obtain Equation (60).

(2) For resonance case (52), we have

(A69) $$H_{0}=\omega_{a}\left(a^{†}a+b^{†}b\right),\;H_{I}=\left(a+a^{†}\right)\left(g^{*}b+gb^{†}\right)+\delta \omega b^{†}b,$$

(A70) $$D_{0}=D_{0}^{†}=\left(gab^{†}+g^{*}a^{†}b\right)+\delta \omega b^{†}b,\;D_{\omega_{a}+\omega_{b}}=D_{-(\omega_{a}+\omega_{b})}^{†}=g^{*}ab.$$

Substituting it in Equation (20), we obtain Equation (61).

As

(A71) $${\langle n_{i}\rangle }_{0}=\frac{1}{e^{\beta \omega_{a}}-1},$$

we have

(A72) $$-{\langle H^{\left(2\right)}\rangle }_{0}={\left|g\right|}^{2}\frac{1-e^{-2\beta \omega_{a}}}{2\omega_{a}}\left(1+{\langle n_{a}\rangle }_{0}\right)\left(1+{\langle n_{b}\rangle }_{0}\right)={\left|g\right|}^{2}\frac{\coth\frac{\omega_{a}\beta }{2}}{2\omega_{a}}.$$

Thus, by Equation (36), we obtain Equation (62).

### Appendix G.3. Two-Level System and Oscillator

(1) For the off-resonance case, we have

(A73) $$H_{0}=\omega_{a}\sigma_{+}\sigma_{-}+\omega_{b}b^{†}b,\;H_{I}=\left(a+a^{†}\right)\left(g^{*}b+gb^{†}\right),$$

(A74) $$D_{\omega_{a}-\omega_{b}}=D_{\omega_{b}-\omega_{a}}^{†}=g\sigma_{-}b^{†},\;D_{\omega_{a}+\omega_{b}}=D_{-(\omega_{a}+\omega_{b})}^{†}=g^{*}\sigma_{-}b$$

and

(A75) $$\begin{array}{cc} D_{\omega_{a}-\omega_{b}}D_{\omega_{a}-\omega_{b}}^{†} & ={\left|g\right|}^{2}\sigma_{-}\sigma_{+}b^{†}b={\left|g\right|}^{2}\left(1-n_{a}\right)n_{b}, \end{array}$$

(A76) $$\begin{array}{cc} D_{\omega_{a}+\omega_{b}}D_{\omega_{a}+\omega_{b}}^{†} & ={\left|g\right|}^{2}\sigma_{-}\sigma_{+}bb^{†}={\left|g\right|}^{2}\left(1-n_{a}\right)\left(n_{b}+1\right), \end{array}$$

(A77) $$\begin{array}{cc} D_{\omega_{b}-\omega_{a}}D_{\omega_{b}-\omega_{a}}^{†} & ={\left|g\right|}^{2}\sigma_{+}\sigma_{-}bb^{†}={\left|g\right|}^{2}n_{a}\left(n_{b}+1\right), \end{array}$$

(A78) $$\begin{array}{cc} D_{-(\omega_{a}+\omega_{b})}D_{-(\omega_{a}+\omega_{b})}^{†} & ={\left|g\right|}^{2}\sigma_{+}\sigma_{-}b^{†}b={\left|g\right|}^{2}n_{a}n_{b}. \end{array}$$

As

(A79) $${\langle n_{a}\rangle }_{0}=\frac{1}{e^{\beta \omega_{a}}+1},\;{\langle n_{b}\rangle }_{0}=\frac{1}{e^{\beta \omega_{b}}-1}$$

for i=a,b, then by Equation (A35), we have

(A80) $$\begin{array}{cc} -{\langle H^{\left(2\right)}\rangle }_{0} & ={\left|g\right|}^{2}\left(\frac{1-e^{-\beta (\omega_{a}-\omega_{b})}}{\omega_{a}-\omega_{b}}\left(1-{\langle n_{a}\rangle }_{0}\right){\langle n_{b}\rangle }_{0}+\frac{1-e^{-\beta (\omega_{a}+\omega_{b})}}{\omega_{a}+\omega_{b}}\left(1-{\langle n_{a}\rangle }_{0}\right)\left(1+{\langle n_{b}\rangle }_{0}\right)\right)\\ \; & ={\left|g\right|}^{2}\frac{\omega_{a}\tanh\frac{\beta \omega_{a}}{2}\coth\frac{\beta \omega_{b}}{2}-\omega_{b}}{\omega_{a}^{2}-\omega_{b}^{2}}. \end{array}$$

By Equation (36), we obtain Equation (66).

(2) For resonance case (52), we have

(A81) $$H_{0}=\omega_{a}\left(\sigma_{+}\sigma_{-}+b^{†}b\right),\;H_{I}=\left(\sigma_{-}+\sigma_{+}\right)\left(g^{*}b+gb^{†}\right)+\delta \omega b^{†}b,$$

(A82) $$D_{0}=D_{0}^{†}=\left(g\sigma_{-}b^{†}+g^{*}\sigma_{+}b\right)+\delta \omega b^{†}b,\;D_{\omega_{a}+\omega_{b}}=D_{-(\omega_{a}+\omega_{b})}^{†}=g^{*}\sigma_{-}b.$$

As

(A83) $${\langle n_{a}\rangle }_{0}=\frac{1}{e^{\beta \omega_{a}}+1},\;{\langle n_{b}\rangle }_{0}=\frac{1}{e^{\beta \omega_{a}}-1},$$

we have

(A84) $$-{\langle H^{\left(2\right)}\rangle }_{0}={\left|g\right|}^{2}\frac{1-e^{-2\beta \omega_{a}}}{2\omega_{a}}\left(1-{\langle n_{a}\rangle }_{0}\right)\left(1+{\langle n_{b}\rangle }_{0}\right)=\frac{{\left|g\right|}^{2}}{2\omega_{a}}.$$

Thus, by Equation (36), we obtain Equation (68).
